# Thermal Characterization of Dynamic Silicon Cantilever Array Sensors by Digital Holographic Microscopy

**DOI:** 10.3390/s17061191

**Published:** 2017-05-23

**Authors:** Marjan Zakerin, Antonin Novak, Masaya Toda, Yves Emery, Filipe Natalio, Hans-Jürgen Butt, Rüdiger Berger

**Affiliations:** 1 Max Planck Institute for Polymer Research, Ackermannweg 10, 55128 Mainz, Germany; zakerin@mpip-mainz.mpg.de (M.Z.); Butt@mpip-mainz.mpg.de (H.-J.B.); 2 Laboratoire d’Acoustique de l’Université du Maine (LAUM, UMR CNRS 6613), 72000 Le Mans, France; antonin.novak@univ-lemans.fr; 3 Graduate School of Engineering, Tohoku University, 6-6-01 Aramaki-Aza-Aoba, Aoba-ku, 980-8579 Sendai, Japan; mtoda@nme.mech.tohoku.ac.jp; 4 Lyncee Tec SA, PSE-A, CH-1015 Lausanne, Switzerland; yves.emery@lynceetec.com; 5 Institut für Chemie, Martin-Luther-Universität Halle-Wittenberg, Kurt-Mothes-Straße 2, 06120 Halle (Saale), Germany; filipe.natalio@chemie.uni-halle.de

**Keywords:** digital holography, micromechanical cantilever sensors, thermal load, temperature coefficient of resonance frequency, temperature coefficient of elastic modulus

## Abstract

In this paper, we apply a digital holographic microscope (DHM) in conjunction with stroboscopic acquisition synchronization. Here, the temperature-dependent decrease of the first resonance frequency (*S*_1_(*T*)) and Young’s elastic modulus (*E*_1_(*T*)) of silicon micromechanical cantilever sensors (MCSs) are measured. To perform these measurements, the MCSs are uniformly heated from *T*_0_ = 298 K to *T* = 450 K while being externally actuated with a piezo-actuator in a certain frequency range close to their first resonance frequencies. At each temperature, the DHM records the time-sequence of the 3D topographies for the given frequency range. Such holographic data allow for the extracting of the out-of-plane vibrations at any relevant area of the MCSs. Next, the Bode and Nyquist diagrams are used to determine the resonant frequencies with a precision of 0.1 Hz. Our results show that the decrease of resonance frequency is a direct consequence of the reduction of the silicon elastic modulus upon heating. The measured temperature dependence of the Young’s modulus is in very good accordance with the previously-reported values, validating the reliability and applicability of this method for micromechanical sensing applications.

## 1. Introduction

In recent years, digital holography (DH) has been widely used for phase-contrast sensing applications both on Earth and in space [1,2,3,4,5,6,7,8,9,10,11,12,13,14,15]. Among different groups, two important studied categories with this technique are biological samples [1,3,4,5,6,7] and micro-components [8,9,10,11,14,15]. In all of these studies, the optically-generated holograms are digitally sampled by a charge-coupled device (CCD) camera. Each hologram is numerically reconstructed as an array of complex numbers, representing the amplitude and phase of the electromagnetic wave field reflected or transmitted by the observed surface. The phase images can be readily applied for the quantitative phase-contrast measurements at any point situated in the field of view for sensing purposes. 

In DH, the phase of the interfering electromagnetic waves which form the holographic interference pattern is determined by the optical path length differences at each point. The optical path length is influenced by: (i) the partial movement of the object in any direction; (ii) the change in the object refractive index; or (iii) the change of the object thickness [16]. By exploiting the first factor, DH can be used for translational, vibrational, or rotational sensing purposes. Moreover, by means of a microscope technique called reflective digital holographic microscopy (R-DHM), DH can be efficiently employed as a full-field technique for sensing the change in the out-of-plane displacement of the diffusively reflective micro-components [9,10,11,12].

So far, the surface displacement of static micromechanical sensors (MCSs) with different shapes and geometries, such as cantilever beams, bridges, and membranes, have been successfully measured upon the heat load [9]. This study has helped researchers to understand the effect of residual stress on the deformation of a single microstructure. Nevertheless, there is a lack of information about the characterization of these structures in the dynamic mode upon heating. The latter is essential since vibrations occur in many Si-based microelectromechanical (MEMS) products, such as mass sensors [17,18,19,20,21], accelerometers [22,23], miniature robots [24], micro-mirrors [25], and transducers [26].

In all of the aforementioned cases, the frequency response of MEMS devices also depends on the temperature. Hence, the corresponding correction factors at different temperatures are very important for reliable data interpretations. Therefore, in this paper we focus on the development of an experimental remote sensing setup based on R-DHM for the systematic measurement of the shift in the resonance frequency of micromechanical cantilever sensors (MCSs) at different temperatures. We follow the first application of the reflective holography to observe the real-time surface vibration modes as reported in 1968 by Archbold and Ennos [8], as well as Watrasiewicz and Spicer [13]. In these first studies, the hologram of a stationary object was recorded with a continuous illumination. Next, the object was excited to vibrate and viewed through the hologram, using a pulsed stroboscopic optical illumination operated at the same excitation frequency. By synchronizing the pulsed stroboscopic optical illumination with the phase of the object movement within the vibration cycle, a quasi-stationary image was obtained. This stationary image corresponded to the deformation of the surface relative to its rest position.

Additionally, the abovementioned measurements inspired us to explore the stroboscopic reflective digital holographic microscope as a new detection technique to measure the in situ dynamic response of several micromechanical cantilever sensors (MCSs) simultaneously. The change of the MCSs resonance frequencies were measured as a function of temperature in the range of *T*_0_ = 298 K to *T* = 450 K. Based on our measurements, we calculate the change of the Young’s modulus of Si upon heating. Advantageously, the interferometric nature of this detection method allows calculating the absolute values of the vibrational amplitudes of MCSs with a sub-nanometric resolution [27].

Such a model system is important to study since different architectures of the MCSs [20,28,29,30,31] have the potential to replace many conventional mass sensors. For the MCS-based mass sensors, the large ratio between an MCS active surface area (in order of 10^−6^ cm^2^) to its mass (nano-grams) leads to both a high sensitivity to surface phenomena and a short response time of sensors (typically μs). Therefore, the MCSs can be used as unique tools for mass sensing both in dynamic and static modes of operation. In the dynamic mode, the MCS resonance frequency varies as a function of mass loading like molecular adsorption, mass loss like molecular desorption, or temperature change.

## 2. Sensing Setup, Data Acquisition and Data Evaluation

**Figure 1:**
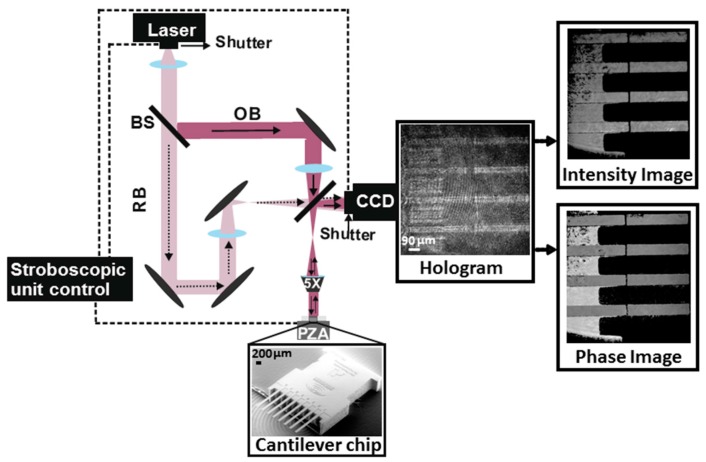
In a R-DHM, a coherent laser beam is split into the OB and the RB. The OB, which is the light reflected off the MCSs interferes with the RB on the CCD camera to form holograms. The phase and intensity images are extracted from the holograms. With the addition of the stroboscopic unit control, the time synchronization between three components of (1) the positions of the piezoelectric actuator (PZA) vibration, (2) the ON-time of the stroboscopic source (laser) pulse, and (3) the CCD camera shutter are controlled. The phase images are used to calculate the amplitudes of the vibrations. The measurements are performed in a closed chamber and through an optical window. The cantilever chip image shown at the bottom as mounted on top of the piezoelectric actuator is taken with a scanning electron microscope.

The experimental set-up is based on a reflective digital holographic microscope (R-DHM) (R1000, Lyncee Tec, Lausanne, Switzerland) (Figure 1). A linearly-polarized laser diode with a wavelength of λ = 689.5 nm is used as the light source. A variable beam splitter divides the laser beam into reference and object beams, respectively. The intensities of the reference and the object beam of the interferometer are adjusted to be similar. The reference beam (RB) illuminates the hologram plane on the CCD camera (Figure 1). The object beam (OB) illuminates the microcantilever array sensor. The light beam back-reflected from the microcantilever interferes with the reference beam at the hologram plane on the CCD camera to create a hologram. When this digitally-recorded hologram is numerically multiplied by the complex amplitude of the same reference beam used to record it, the 3D topography of the cantilever array sensor is reconstructed with the interferometric vertical resolution (typically 0.3 nm with the current setup). By using Fourier filtering analysis, resolution down to a few pico-meters can be achieved.

The optical microscope used for this experiment, inserted into the object beam path, has an objective with 5× magnification. Its numerical aperture and working distance are 0.12 and 14 mm, respectively, resulting in a measurement with the lateral resolution of 2.85 μm. With such a setup, 3D images of MCSs can be routinely recorded in real-time. In the case of MCSs, the R-DHM measures a length of *l* = 485.94 ± 2.85 μm and a width of *w* = 90 ± 2.85 μm, respectively. The nominal thickness of the cantilever sensors, provided by the manufacturer (Micromotive GmbH, Mainz, Rhineland-Palatinate, Germany) is *h* = 5.0 ± 0.3 μm. Since we use a 5× objective, four out of eight cantilever sensors lie in the field of view (Figure 1). We refer the interested reader to the paper of Cuche et al. for the detailed explanation of the digital recording and reconstruction of the holograms [27].

MCSs are often operated in the dynamic mode, i.e., the vibrational response of the device changes upon a recognition event, such as the change of the mechanical property or the mass. For MCSs a recognition event can induce changes in its vibrational amplitude upon the shift in the resonance frequency. In order to measure the amplitude of the out-of-plane vibration of the MCSs, the R-DHM is operated in combination with a stroboscopic control unit. The stroboscopic unit is connected to a piezoelectric actuator (PZA) to externally excite the out-of-plane vibrations of the cantilever. In addition, the stroboscopic unit controls the time synchronization between the positions of the PZA vibration, the ON-time of the stroboscopic source (laser) pulse which is made by the introduction of a shutter in front of the laser and the CCD camera shutter (Figure 1). In the following paragraph we explain the sampling of the MCS vibrational amplitude within one period of vibration for each desired frequency with such a setup.

**Figure 2:**
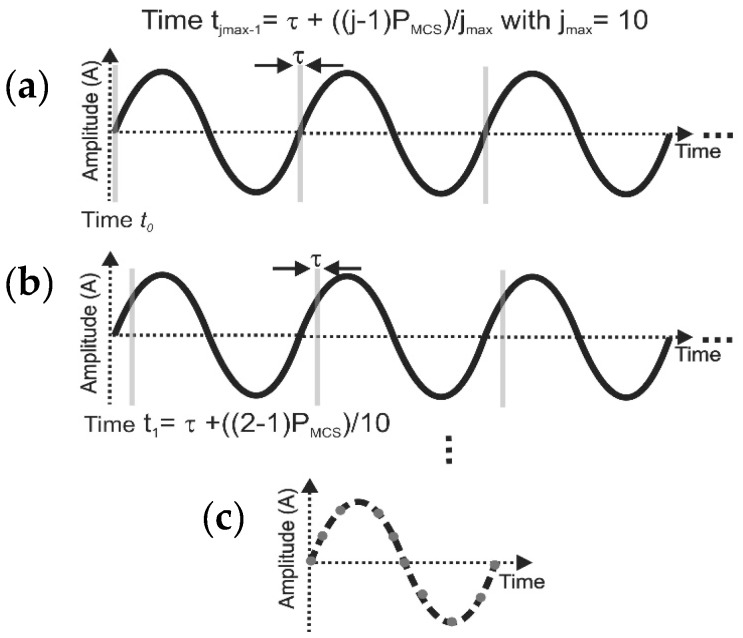
(**a**) As an illustrative example, three periods of a cantilever vibration are plotted in a diagram of amplitude (A) versus time. For a typical driving frequency applied to the cantilever, *f_MCS_*, the period of movement is calculated as *P_MCS_* = 1/*f_MCS_*. (**b**) For each *P_MCS_*, the piezo-actuator excites the cantilevers periodically while a laser pulse with the pulse length time of τ = 167.5 ns is shifted in time with respect to the excitation signal to sample *j_max_* (here 10) number of different moments (*t_j_*_−1_). (**c**) For each moment, the signals from 2074 pulses are integrated by keeping the CCD camera shutter open (Figure 1). Finally, by fitting a sine function (dash line) to the *j_max_* = 10 desired measured moments or the so-called samples/period (dots), the vibrational amplitude for any arbitrary period is reconstructed.

Exemplarily, the amplitude, A, of the cantilever movement is plotted versus time for one period, *P_MCS_* (s), as shown in Figure 2a. The illuminated laser pulse has a width of *τ* and is preliminarily initiated at time *t*_0_. To measure the cantilever’s out-of-plane amplitude within one period, the much shorter laser pulse of *τ* (*τ* << *P_MCS_*) is shifted in time with respect to the excitation signal as:

(1) $$t_{j-1}=\tau +\frac{\left(j-1\right)P_{MCS}}{j_{max}}$$

where, *j* = 1, 2, 3, …, *j_max_* with *j_max_* is number of desired sampled moments by the laser pulse within one period (or simply sample/period) and *t_j_*_−1_ indicates the sampling moment within the period of the vibration. In our setup, the electronics enable us to synchronize the timings of the CCD camera shutter, the laser pulse with a minimum width of *τ* = 7.5 ns, and the PZA. In order to increase the signal-to-noise ratio of the digitally-recorded holograms on the CCD camera, the recorded hologram at each vibration moment, *t_j_*_−1_, is integrated over a specific number of pulses (or, consequently, over a specific number of periods). The latter is indicated by the vertical grey lines (Figure 2a). Therefore, an additional shutter is mounted in front of the CCD camera to adjust the integration time (Figure 1). Doing so, enough signals are obtained for producing high-quality holograms. The number of pulses are determined by integration time/pulse length time. In our measurements, for each *t_j_*_−1_, the static shutter is set to 453 μs to add the intensity of 2074 pulses with the pulse length time of *τ* = 167.5 ns.

The operating software of the R-DHM (Koala, Lyncee Tec, Lausanne, Switzerland) computes the intensity and phase images from the recorded holograms. In the recorded phase images, the absolute value of the vibrational amplitude of the MCS, Δ*A*, is directly proportional to the difference between the optical path lengths of the laser pulse wavefronts (see above) reflected off: (a) the vibrating cantilevers; and (b) a fixed point/area on the base of the cantilever array sensor. For a homogeneous sample consisting of only one material, Δ*A* value is directly calculated from the value of Δ*φ* with the help of the following equation [9]:

(2) $$\Delta A=\frac{\lambda \Delta \phi }{4\pi n}$$

Here, *n* is the refractive index of the immersion medium (here *n* = 1 for air), and *λ* is the wavelength of the laser source (here *λ* = 689.5 nm). 

In our experiments, the cantilevers are excited externally with a piezo-actuator (AE0203D04F, Thorlabs, Munich, Bavaria, Germany) using a sine wave. In order to measure the first natural resonance frequencies of the MCSs, the piezo-actuator frequency is scanned with the discrete steps of 5 ± 0.1 Hz from 29.4000 to 30.0000 kHz. The frequency resolution of 0.1 Hz is the precision of the waveform generator in the stroboscopic unit control for the full frequency range.

The so-called MEMSTool analysis tool package (Lyncee Tec, Lausanne, Switzerland) was used to calculate the absolute values of the vibrational amplitudes, Δ*A*, of MCSs from the Δ*φ* values. Δ*φ* values are evaluated from the recorded phase images (Equation (2)).

**Figure 3:**
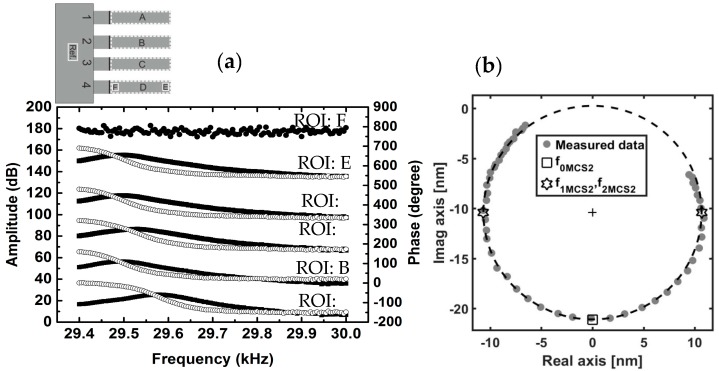
(**a**) Bode graph for six different ROIs on the MCSs at *T* = 298 K. At the resonance frequency of *f*_0*MCSs*_, the cantilever response is in phase quadrature (i.e., *θ* = −π/2) with the excitation signal. On the vertical axes on left- and right-hand sides, the amplitude and phase values for A, B, C, D, and E ROIs are shifted by the values of 30 dB and 160 degrees with respect to each other, respectively. For ROI F, the amplitude values are shifted by the value of 60 dB. The region shown as Ref. is the reference area; (**b**) The real-imaginary Nyquist plane of the experimental data for MCS2 from the ROI B (gray dots) with the circle fit (dashed black line). The evaluated resonance frequency *f*_0*MCS*2_ at *θ* = −π/2 (square) was determined with 0.1 Hz precision as *f*_0*MCS*2_ = 29.4946 kHz. The quality factor is also calculated by evaluating the bandwidth, Δ*f_MCS_*_2_ = *f*_2*MCS*2_ − *f*_1*MCS*2_ (hexagrams). In this case, the quality factor is $Q_{MCS2}=\frac{f_{0MCS2}}{f_{2MCS2}-f_{1MCS2}}=\frac{29.4946}{29.5530-29.4321}\;=$ 243.9946 ± 0.0001.

By choosing the desired region of interests (ROIs) on the phase images, the results can be visualized in two different ways. First, in the form of Bode graphs (Figure 3a), depicting the amplitude (in dB relative to 1 nm) and the phase of the externally excited MCSs response (displacement) as a function of frequency [32]. Second, in the form of Nyquist graphs (Figure 3b) in which the imaginary part of the transfer function is plotted as a function of its real part [33]. The following two parameters are then estimated for each set of measured data: (i) the resonance frequency *f*_0*MCS*_ and (ii) the quality factor *Q_MCS_* which is defined as:

(3) $$Q_{MCS}=\frac{f_{0MCS}}{f_{2MCS}-f_{1MCS}}$$

Here, *f*_2*MCS*_ and *f*_1*MCS*_ are the lower and higher frequencies at which the amplitude of the micromechanical cantilever vibration drops by −3 dB relative to the maximum values. 

In this study, the resonance frequency *f*_0*MCS*_ and the quality factor *Q_MCS_* could be estimated directly from reading the Bode plots (Figure 3a). However, since the measurements are carried out with the discrete steps of 5 ± 0.1 Hz in the presence of the inevitable mechanical noise from the environment, such an approach may lead to an error in the order of the discrete step size, i.e., 5 Hz in our case. Under such conditions, the circle-fitting algorithm is a much more accurate and very often used modal analysis method [34]. 

The circle-fit method takes the advantage of the fact that the frequency response function of a single-degree-of-freedom (SDOF) system, when plotted on the real-imaginary Nyquist plane, is a circle [33]. This method, although developed for single-degree-of-freedom systems, can be used for separate modes of the multiple-degree-of-freedom systems, such as the MCSs vibrating in their first mode [33,34]. The circle-fitting algorithm consists of drawing the best circle possible through the measured points around the resonance frequency using a least squares circle fit (Figure 3b). Furthermore, it allows one to estimate the possible time delay introduced by the latency of the measurement chain by centering the circle to its canonical position as well as setting *f*_0*MCS*_, *f*_1*MCS*_, and *f*_2*MCS*_ at $-\frac{\pi }{2}$, $-\frac{\pi }{4}$, and $-\frac{3\pi }{4}$, respectively [32,33,34]. By using this method, the resonance frequencies of MCSs can be evaluated with accuracy even better than the precision of the waveform generator of the stroboscopic unit, which was 0.1 Hz. However, we limit the accuracy of our data evaluation with the circle-fit algorithm to match the precision of the waveform generator.

Figure 3a shows the Bode graphs for six selected ROIs, specified with A–F on the cantilever array sensor at *T*_0_ = 298 K. The ROIs labeled with A, B, C, and D include all of the moving parts of each MCS. The E- and F-labeled ROIs, however, are selected within the D region on MCS number 4 at the tip and very close to the clipping part, respectively. For all selected ROIs, the plot of the amplitude versus frequency characterizes the system’s response to different input frequencies. By fitting the circle on the experimental data on the real-imaginary Nyquist plane (as shown in Figure 3b), the resonance frequencies of the MCSs, as well as the quality factors for all of the regions A–D were determined with the precision of 0.1 Hz. The results are presented in Table 1.

We calculated the resonance frequency of a typical MCS with:

(4) $$f_{0MCS\_Cal}\left(T\right)=\frac{1}{2\pi }\sqrt{\frac{k\left(T\right)}{0.24m}}$$

For a comparison against the measured values. Here, *m* is the mass of the cantilever. The spring constant of a rectangular MCS, *k*(*T*) at a given temperature can be calculated from:

(5) $$k\left(T\right)=\frac{E\left(T\right)wh^{3}}{4l^{3}}$$

where *E*(*T*) is the temperature-dependent Young’s modulus of the cantilever material. *w*, *h*, and *l* are the width, the thickness, and the length of the cantilever, respectively. For typical values of *E* = 169 GPa for silicon, *l* = 485.9400 µm, *w* = 90.0000 µm, *h* = 5.0000 µm for a MCS, one obtains *k*(*T*) = 4.1420 N/m. The volume of the MCS is additionally calculated to be *V* = 2.2 × 10^−7^ cm^3^. Next, by taking into account the density of silicon to be *ρ* = 2.331 g/cm^3^, the mass of the typical MCS is calculated to be (0.24*m*) = 5.1 × 10^−10^ kg. By inserting the values of *k*(*T*) and *m* into Equation (4), the calculated resonance frequency is *f*_0*MCS_Cal*_ = 29.1243 kHz. For simplicity, we assume that *l* and *w* are constant, but the nominal value of the thickness *h* = 5 µm might vary by up to the value of ±Δ*h*. In this case, the measured resonance frequencies suggest the variation of Δ*h*_1_ = 74.4 nm, Δ*h*_2_ = 63.6 nm, Δ*h*_3_ = 69.1 nm, and Δ*h*_4_ = 68.3 nm in the thicknesses of the MCSs, respectively. These values are in the expected range of the Δ*h* = ±300 nm for the thickness variation, reported by the manufacturer (Micromotive GmbH, Mainz, Rhineland-Palatinate, Germany). Further, the evaluated value of *f*_0*MCS*4_ for both E and D regions are equal with each other (*f*_0*MCS*4_ = 29.4959 kHz). This, in return, suggests that selecting only a small ROI, like region E, at the tip of each MCS is enough for the evaluation of the resonance frequencies. In region F, however, the Bode graph shows only a relative movement with low amplitude and without a resonance peak. We expect such a behavior since this ROI is next to the clamped part of the MCS number 4 cantilever.

## 3. Evaluation of the Temperature Coefficient of Resonance Frequencies of Heated MCSs

**Figure 4:**
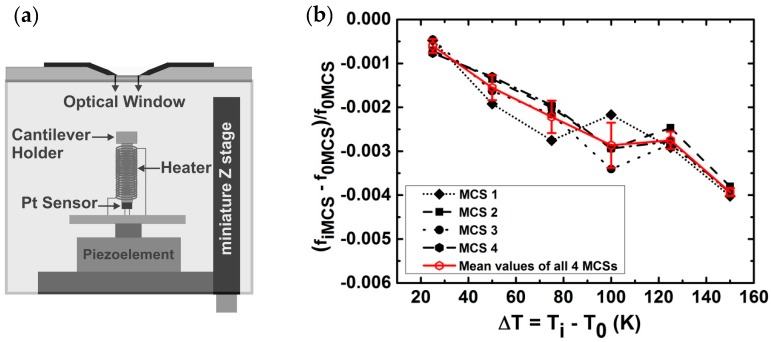
(**a**) The schematic shows the experimental chamber used for the uniform heating of the MCSs in quasi-sealed condition. The schematic is not to scale; (**b**) For each MCS (in black) and their corresponding mean values (in gray) the measured values of $\frac{\partial f}{f_{0MCS}}=\frac{f_{iMCS}-f_{0MCS}}{f_{0MCS}}$ is plotted versus Δ*T = T_i_* − *T*_0_. Here, *f*_0*MCS*_ is the initial resonance frequency measured at *T*_0_ = 298 K and *f_iMCS_* is the resonance frequency measured at desired temperatures of *T_i_* during the uniform heating from *T*_0_ = 298 K to *T* = 450 K. A fit on the mean values graph of all four cantilevers yields the mean slope of $S_{1}\left(T\right)=\frac{\partial f}{\Delta Tf_{0MCS}}=-25.75\;\pm \;1.94\;\times 10^{-6}\;K^{-1}$ with the intersection value of −0.0039 × $10^{-6}$ with the vertical axis. All resonance frequency values are evaluated from the circle fit method with the precision of 0.1 Hz.

In the next step, the MCSs out-of-plane amplitudes are recorded during the frequency sweeps at different temperatures, ranging from *T*_0_ = 298 ± 0.1 K to *T* = 450 ± 0.1 K, with temperature steps of 25 K. The cantilevers are uniformly heated and the temperature is controlled with a home-built oven (Figure 4a). The heater is a low- and constant-resistance solenoid around a hallow brass cantilever holder. The temperature is read out from the resistance change of a platinum resistive (Pt) sensor, positioned in the hallow brass cylinder inside the solenoid. The miniature Z-stage provides the fine distance adjustments for the hologram formation.

When the silicon MCSs are heated, their resonance frequencies decrease. This decrease arises due to (1) a decrease in the value of Young’s modulus of silicon with increasing temperature; and (2) a geometrical dimension increase of the MCSs as the result of the thermal expansion of Si. The relative change of the resonance frequency with the temperature can be written as [35]:

(6) $$\frac{1}{f_{0MCS}}\frac{\partial f\left(T\right)}{\partial T}=\frac{1}{2k}\frac{dk\left(T\right)}{dT}$$

where, again, *f*_0*MCS*_ is the resonance frequency of a MCS, $f\left(T\right)=\frac{1}{2\pi }\sqrt{\frac{k\left(T\right)}{0.24m}}$, $k\left(T\right)=\frac{E\left(T\right)wh^{3}}{4l^{3}}$, and *T* is the temperature. In fact, both of the above mentioned factors are included in the change of the spring constant, *k*(*T*), of the MCSs with the temperature. By substituting Equation (5) into Equation (6) one obtains [35]:

(7) $$\frac{1}{f_{\left(0MCS,\;T_{0}\right)}}\frac{\partial f\left(T\right)}{\partial T}=\frac{1}{2}\left(\frac{1}{k}\frac{dk\left(T\right)}{dT}\right)=\frac{1}{2}\left(\frac{1}{E\left(T_{0}\right)}\frac{dE}{dT}+\frac{1}{w\left(T_{0}\right)}\frac{dw}{dT}+\frac{3}{h\left(T_{0}\right)}\frac{dh}{dT}-\frac{3}{l\left(T_{0}\right)}\frac{dl}{dT}\right)$$

The subscript zero indicates the variable at the initial temperature of *T*_0_ = 298 K. Additionally, we know that the isotropic thermal expansion coefficient is given by [35]:

(8) $$\alpha =\frac{1}{w\left(T_{0}\right)}\frac{dw}{dT}=\frac{1}{h\left(T_{0}\right)}\frac{dh}{dT}=\frac{1}{l\left(T_{0}\right)}\frac{dl}{dT}$$

Inserting Equation (8) in Equation (7) gives:

(9) $$\frac{1}{f_{\left(0MCS,\;T_{0}\right)}}\frac{\partial f\left(T\right)}{\partial T}=\frac{1}{2}\left(\frac{1}{k}\frac{dk\left(T\right)}{dT}\right)=\frac{1}{2}\left(\frac{1}{E\left(T_{0}\right)}\frac{dE\left(T\right)}{dT}+\alpha \right)$$

Here, the term $\frac{1}{f_{\left(0MCS,\;T_{0}\right)}}\left(\frac{\partial f}{\partial T}\right)$ $=S_{1}\left(T\right)$ is the first temperature coefficient of the frequency. In addition, $\frac{1}{E\left(T_{0}\right)}\frac{dE}{dT}=E_{1}\left(t\right)$ is the first temperature coefficient of the Young’s elastic modulus showing the decrease of elastic modulus upon heating. Now we can rewrite Equation (9) as:

(10) $$S_{1}\left(T\right)=\frac{1}{f_{\left(0MCS,\;T_{0}\right)}}\frac{\partial f\left(T\right)}{\partial T}=\frac{1}{2}\left(\frac{1}{E\left(T_{0}\right)}\frac{dE}{dT}+\alpha \left(T\right)\right)=\frac{1}{2}(E_{1}\left(T\right)+\alpha \;)$$

It is noteworthy to mention that since Si properties exhibit an almost linear dependence upon the increase of temperature in the range of *T*_0_ = 298 K to *T* = 450 K, only the first-order terms are used in Equation (9). The goal is to determine which term on the right-hand side of Equation (9) is the dominating term for the decrease of the resonance frequencies of MCSs upon heating. To do so, we first need to obtain the $S_{1}\left(T\right)$ values. This is simply done by plotting our experimental data in the form of $\partial f/f_{0MCS}=f_{iMCS}-f_{0MCS}/f_{0MCS}$ versus Δ*T* = *T_i_* − *T*_0_ (Figure 4b). Here, *f*_0*MCS*_ is the value of the resonance frequency at *T*_0_ = 298 K and *f_iMCS_* is the resonance frequency measured at the given temperatures of *T_i_*. The tangent of a line fitted to each graph yields: $S_{1}\left(T\right)=\partial f/(\Delta Tf_{0MCS})$. The $S_{1}\left(T\right)$ values for the MCSs labelled with the numbers 1–4 are: −23.0 ± 5.3 × 10^−6^ K^−1^, −22.6 ± 3.7 × 10^−6^ K^−1^, −25.5 ± 4.5 × 10^−6^ K^−1^, and −24.4 ± 2.7 × 10^−6^ K^−1^, respectively. The mean value of *S*_1_(*T*) for all four cantilevers is 25.75 ± 1.94 × 10^−6^ K^−1^. These values are in perfect agreement with the former reported values for Si(100), lying between −23.6 × 10^−6^ K^−1^ and −26.6 × 10^−6^ K^−1^ [36]. The reported range of values for the isotropic thermal expansion coefficient of Si in the temperature range of *T*_0_ = 298 K to *T* = 450 K is *α* = 2.555 × 10^−6^ K^−1^ to 3.453 × 10^−6^ K^−1^ [37]. By taking the value of *α*(*T =* 450 K) = 3.453 × 10^−6^ K^−1^ for pure Si at the maximum measured temperature of *T_i_* = 450 K, with a precision of 10^−8^ K^−1^ [37], the first temperature coefficient of the elastic modulus, $E_{1}\left(T\right)$, can be easily calculated. To perform the calculation, we re-write Equation (9).

(11) $$E_{1}\left(T\right)=2S_{1}\left(T\right)-\alpha \left(T=450\;K\right)$$

which, in return, results in a value of $-54.95\pm 3.88\;\times 10^{-6}\;K^{-1}$ for all four Si cantilever sensors in the range of 298–450 K. This value is in very good agreement with the previously reported values of −52.6 ± 3.45 × 10^−6^ K^−1^ for the temperature range of 200–290 K [36]. Additionally, the value of *α*(*T =* 450 K) = 3.453 × 10^−6^ K^−1^ is only 6% of the value of $E_{1}\left(T\right)$ = $-54.\;95\pm 3.88\;\times 10^{-6}\;K^{-1}$ for Si. Therefore, the decrease of the Young’s modulus of Si upon heating has the dominant contribution in the decrease of *S*_1_ *(T)* value versus temperature and, consequently, in the reduction of the resonance frequency with the increase of temperature. In this case if one neglects the effect of Si thermal expansion, then $E_{1}\left(T\right)\approx 2S_{1}\left(T\right)\approx -51.50\pm 3.88\;\times 10^{-6}\;K^{-1}.$

For the MCSs labelled 1–4, the decrease of the resonance frequency upon heating from *T*_0_ = 298 K to *T* = 450 ± 0.1 K is equal to Δ*f_MCS_*_1_ *=* (*f*_450 *K*_ − *f*_298 *K*_)*_MCS_*_1_ = −119.1 ± 0.1 Hz, Δ*f_MCS_*_2_ *=* (*f*_450 *K*_ − *f*_298 *K*_)*_MCS_*_2_ = −113.3 ± 0.1, Δ*f_MCS_*_3_ = (*f*_450 *K*_ − *f*_298 *K*_)*_MCS_*_3_ = −118.5 ± 0.1 Hz, and Δ*f_MCS_*_4_ = (*f*_450 *K*_ − *f*_298 *K*_)*_MCS_*_4_ = −117.2 ± 0.1 Hz where the subscript number shows the MCS‘s number. Moreover, in these measurements the quality factor shows less than 2% of change when the temperature changes from *T*_0_ = 298 K to *T* = 450 K ± 0.1 K. Therefore, they are not discussed further. 

## 4. Summary and Conclusions

In this paper, a stroboscopic reflective-DHM was used to measure the simultaneous out-of-plane vibrational amplitudes of four externally-actuated MCSs operating in a temperature range of *T*_0_ = 298 K to *T* = 450 K. Our experiments have demonstrated successful remote sensing measurements of: (a) the shift in the resonance frequency; and (b) the reduction of the Young’s elastic modulus of the MCSs upon heating.

One of the key advantageous of the DHM is the 3D recording of the information over the full field of view with only one single hologram acquisition, and without any lateral or vertical scanning of the laser light beam. Therefore our technique realizes a readout which is not affected by lateral drift with regard to changes in temperature. Moreover, advantageously, our laterally-resolved holographic images do not experience crosstalk effects between the adjacent cantilever sensors. For an example of this type of crosstalk, we refer the reader to [38] in which parts of the laser beam hit adjacent structures in a way that results in artifacts when reading out the movement of the cantilever sensors. 

Finally, by plotting $\frac{\partial f}{f_{0}}$ versus Δ*T* = *T_i_* − *T*_0_ and fitting a line to the experimental data, we have evaluated the values of *S*_1_(*T*) and consequently determined the values of *E*_1_(*T*) as described in Section 3*.* For the calculation of *E*_1_(*T*) value, the isotropic expansion of silicon microcantilever sensors was taken into account (Equation (11)). In particular, to avoid the deformation of the micromechanical cantilever sensors by the thermal stress, the cantilever array sensor was heated with a rate of 1 K/s until the desired temperature was reached. Under these conditions, we can safely assume that the silicon MCSs expand isotropically upon the heat load. Our results demonstrate that the reduction of Young’s modulus upon heating is the dominant effect for the decrease of the natural resonance frequency of MCSs upon heating. With regard to the applications of our technique, although the expansion of cantilevers is isotropic, our sensing setup based on stroboscopic reflective-DHM can also be readily applied to study systems composed of different materials which expand non-isotropically upon thermal load. Examples include the effect of thermo-mechanical loads in: (a) the IC-fabrication and optimization process; and (b) the manufacturing and packaging of complex MEMS and micro-components [14,15]. Furthermore, the results also indicate that our technique can be reliably used for future mass sensing applications at elevated temperatures, which is required, for example, for the thermogravimetric analysis of minute amounts of materials. 

## Figures and Tables

**Table 1:** The measured resonance frequency *f*_0_ of each micromechanical cantilever sensor (MCS) at the reference temperature of *T*_0_ = 298 K together with their corresponding quality factors, Q_MCS_.

MCS	1	2	3	4
*f*_0*MCS*_ (kHz) ± 0.0001	29.5777	29.4946	29.5267	29.4959
*Q_MCS_*	243.9946	242.2243	214.8007	237.4964

## References

[1] Khmaladze A., Kim M., Lo C.-M. (2008). Phase imaging of cells by simultaneous dual-wavelength reflection digital holography. Opt. Express.

[2] Khmaladze A., Restrepo-Martínez A., Kim M., Castañeda R., Blandón A. (2008). Simultaneous dual-wavelength reflection digital holography applied to the study of the porous coal samples. Appl. Opt..

[3] Rappaz B., Barbul A., Hoffmann A., Boss D., Korenstein R., Depeursinge C., Magistretti P.J., Marquet P. (2009). Spatial analysis of erythrocyte membrane fluctuations by digital holographic microscopy. Blood Cells Mol. Dis..

[4] Mendoza F., Aguayo D.D., Manuel H., Salas-Araiza M.D. (2011). Butterflies’ wings deformations using high speed digital holographic interferometry. Proc. SPIE.

[5] Khmaladze A., Matz R.L., Epstein T., Jasensky J., Banaszak Holl M.M., Chen Z. (2012). Cell volume changes during apoptosis monitored in real time using digital holographic microscopy. J. Struct. Biol..

[6] Toy M.F., Richard S., Kühn J., Franco-Obregón A., Egli M., Depeursinge C. (2012). Enhanced robustness digital holographic microscopy for demanding environment of space biology. Biomed. Opt. Express.

[7] Roy M., Seo D., Oh S., Yang J.-W., Seo S. (2017). A review of recent progress in lens-free imaging and sensing. Biosens. Bioelectron..

[8] Archbold E., Ennos A.E. (1968). Observation of surface vibration modes by stroboscopic hologram interferometry. Nature.

[9] Coppola G., Ferraro P., Iodice M., Nicola S.D., Finizio A., Grilli S. (2004). A digital holographic microscope for complete characterization of microelectromechanical systems. Meas. Sci. Technol..

[10] Ferraro P., Coppola G., Nicola S.D., Finizio A., Grilli S., Iodice M., Magro C., Pierattini G. Digital holography for characterization and testing of mems structures. Proceedings of the IEEE/LEOS International Conference on Optical MEMs.

[11] Seebacher S., Osten W., Baumbach T., Jüptner W. (2001). The determination of material parameters of microcomponents using digital holography. Opt. Lasers Eng..

[12] Jueptner W.P.O., Kujawinska M., Osten W., Salbut L.A., Seebacher S. (1998). Combined measurement of silicon microbeams by grating interferometry and digital holography. Int. Soc. Opt. Photonics.

[13] Watrasiewicz B.M., Spicer P. (1968). Vibration analysis by stroboscopic holography. Nature.

[14] Grosser V., Bombach C., Faust W., Vogel D., Michel B. Optical measurement methods for mems applications. Proceedings of the International Conference on Applied Optical Metrology 340.

[15] Pagliarulo V., Miccio L., Ferraro P. (2016). Digital holographic microscopy for the characterization of microelectromechanical systems. Proc. SPIE.

[16] Kim M.K. (2010). Principles and techniques of digital holographic microscopy. J. Photonics Energy.

[17] Thundat T., Wachter E.A., Sharp S.L., Warmack R.J. (1995). Detection of mercury vapor using resonating microcantilevers. Appl. Phys. Lett..

[18] Berger R., Lang H.P., Gerber C., Gimzewski J.K., Fabian J.H., Scandella L., Meyer E., Güntherodt H.J. (1998). Micromechanical thermogravimetry. Chem. Phys. Lett..

[19] Takahito O., Masayoshi E. (2004). Mass sensing with resonating ultra-thin silicon beams detected by a double-beam laser doppler vibrometer. Meas. Sci. Technol..

[20] Toffoli V., Carrato S., Lee D., Jeon S., Lazzarino M. (2013). Heater-integrated cantilevers for nano-samples thermogravimetric analysis. Sensors.

[21] Torres F., Uranga A., Riverola M., Sobreviela G., Barniol N. (2016). Enhancement of frequency stability using synchronization of a cantilever array for mems-based sensors. Sensors.

[22] Weigold J.W., Najafi K., Pang S.W. (2001). Design and fabrication of submicrometer, single crystal si accelerometer. J. Microelectromech. Syst..

[23] Ang W.T., Khoo S.Y., Khosla P.K., Riviere C.N. Physical Model of a MEMS Accelerometer for Low-g Motion Tracking Applications. Proceedings of the 2004 IEEE International Conference on Robotics and Automation, ICRA ’04.

[24] Donald B.R., Levey C.G., McGray C.D., Paprotny I., Rus D. (2006). An untethered, electrostatic, globally controllable MEMS micro-robot. J. Microelectromech. Syst..

[25] Juan W.H., Pang S.W. (1996). Controlling sidewall smoothness for micromachined Si mirrors and lenses. J. Vac. Sci. Technol. B Microelectron. Nanometer Struct. Process. Meas. Phenom..

[26] Qiu Y., Gigliotti J., Wallace M., Griggio F., Demore C., Cochran S., Trolier-McKinstry S. (2015). Piezoelectric Micromachined Ultrasound Transducer (PMUT) Arrays for Integrated Sensing, Actuation and Imaging. Sensors.

[27] Cuche E., Marquet P., Depeursinge C. (1999). Simultaneous amplitude-contrast and quantitative phase-contrast microscopy by numerical reconstruction of Fresnel off-axis holograms. Appl. Opt..

[28] Etayash H., Khan M.F., Kaur K., Thundat T. (2016). Microfluidic cantilever detects bacteria and measures their susceptibility to antibiotics in small confined volumes. Nat. Commun..

[29] Fabian J.-H., Berger R., Lang H.P., Gerber C., Gimzewski J.K., Gobrecht J., Meyer E., Scandella L. Micromechanical thermogravimetry on single zeolite crystals. Proceedings of the uTAS ’98 Workshop Micro Total Analysis Systems ’98.

[30] Iervolino E., van Herwaarden A.W., van der Vlist W., Sarro P.M. Thermogravimetric device with integrated thermal actuators. Proceedings of the 2010 IEEE 23rd International Conference on Micro Electro Mechanical Systems (MEMS).

[31] Berger R., Delamarche E., Lang H.P., Gerber C., Gimzewski J.K., Meyer E., Güntherodt H.-J. (1997). Surface stress in the self-assembly of alkanethiols on gold. Science.

[32] De Silva C.W. (2006). Vibration: Fundamentals and Practice.

[33] Kennedy C.C., Pancu C.P.D. (1947). Use of vectors in vibration measurement and analysis. J. Aeronaut. Sci..

[34] Ewins D.J. (2000). Modal Testing: Theory and Practice.

[35] Ziegler C. (2004). Cantilever-based biosensors. Anal. Bioanal. Chem..

[36] Boyd E.J., Li L., Blue R., Uttamchandani D. (2013). Measurement of the temperature coefficient of Young’s modulus of single crystal silicon and 3c silicon carbide below 273 K using micro-cantilevers. Sens. Actuators A Phys..

[37] Swenson C.A. (1983). Recommended values for the thermal expansivity of silicon from 0 to 1000 K. J. Phys. Chem. Ref. Data.

[38] Lang H.P., Berger R., Andreoli C., Brugger J., Despont M., Vettiger P., Gerber C., Gimzewski J.K., Ramseyer J.P., Meyer E. (1998). Sequential position readout from arrays of micromechanical cantilever sensors. Appl. Phys. Lett..

